# Resveratrol Inhibits Ischemia-Induced Myocardial Senescence Signals and NLRP3 Inflammasome Activation

**DOI:** 10.1155/2020/2647807

**Published:** 2020-08-25

**Authors:** Hong Feng, Shan-qi Mou, Wen-jing Li, Nan Zhang, Zi-ying Zhou, Wen Ding, Zhou-Yan Bian, Hai-han Liao

**Affiliations:** ^1^Department of Geriatrics, Renmin Hospital of Wuhan University, Wuhan, Hubei 430060, China; ^2^Department of Cardiology, Renmin Hospital of Wuhan University, Wuhan, 430060 RP, China; ^3^Hubei Key Laboratory of Metabolic and Chronic Diseases, Wuhan, 430060 RP, China

## Abstract

**Aims:**

The aim of this study was to investigate whether resveratrol (RSV) could ameliorate ischemia- and hypoxia-associated cardiomyocyte apoptosis and injury via inhibiting senescence signaling and inflammasome activation.

**Materials and Methods:**

Mice were treated with RSV by gastric tube (320 mg/kg/day) or vehicle one week before left coronary artery ligation or sham surgery until the end of the experiments. After pressure–volume loop analysis, mouse hearts were harvested for histopathological (including PSR, TTC, TUNEL staining, immunohistochemistry, and immunofluorescence) and molecular analysis by western blotting and RT-PCR. In addition, neonatal rat cardiomyocytes (NRCMs), cardiac fibroblasts (CFs), and macrophages were isolated for *in vitro* experiments. *Key Findings*. RSV treatment decreased mortality and improved cardiac hemodynamics. RSV inhibited the expression of senescence markers (p53, p16, and p19), inflammasome markers (NLRP3 and Cas1 p20), and nuclear translocation of NF-*κ*B, hence alleviating infarction area, fibrosis, and cell apoptosis. RSV also inhibited expression of interleukin- (IL-) 1*β*, IL-6, tumor necrosis factor-*α*, and IL-18 *in vivo*. In *in vitro* experiment, RSV prevented hypoxia-induced NRCM senescence and apoptosis. After inhibition of sirtuin 1 (Sirt1) by EX27, RSV failed to inhibit p53 acetylation and expression. Moreover, RSV could inhibit expression of NLRP3 and caspase 1 p20 in NRCMs, CFs, and macrophages, respectively, in in vitro experiments. *Significance*. Our findings revealed that RSV protected against ischemia-induced mouse heart injury in vivo and hypoxia-induced NRCM injury in vitro via regulating Sirt1/p53-mediated cell senescence and inhibiting NLRP3-mediated inflammasome activation.

## 1. Introduction

Ischemic heart disease (IHD), including acute and stable IHD, remains one of the most burdensome health problems worldwide [1]. Common characteristics of IHD are the obstruction or narrowing of arteries and arterioles resulted in inadequate supply of oxygenated blood for the myocardium [1]. Persistent myocardial ischemia and hypoxia will cause malignant cardiac remodeling and subsequent heart failure. Inhibiting malignant cardiac remodeling has been considered an efficient strategy for preventing the occurrence and development of heart failure.

The molecular mechanisms of cardiac remodeling during IHD are complex [2–4]. Cellular senescence is one of the main mechanisms involved [5, 6]. The p53-associated signaling pathway has been demonstrated to regulate hypoxia-induced apoptosis and senescence [5]. In ischemic heart tissue, senescence-associated *β*-galactosidase (SA-*β*-gal) activity and proteins (such as p16, p19, and p53) are significantly upregulated [6]. Acute or chronic inflammation induced by ischemia has also been suggested to play an important role in the process of malignant cardiac remodeling [3]. In recent years, many studies have shown that NACHT, LRR, and PYD domain-containing protein 3 (NLRP3) inflammasome play a pivotal role in the initiation and progression of IHD and cardiac remodeling [3]. Mechanistically, the inflammasome promotes the activation of caspase 1 and the secretion and accumulation of proinflammatory cytokines such as interleukin- (IL-) 1*β* and IL-18 [3]. Inhibiting the NLRP3 pathway has been suggested to be an effective strategy for protecting against IHD [7].

Resveratrol (RSV) possesses many therapeutic effects for a variety of diseases [8] and is suggested to be a potential promoter of longevity. Previous studies showed that RSV was a specific agonist of sirtuin 1 (Sirt1) [9]. RSV has also been found to regulate other molecular targets such as AMP-activated protein kinase, nuclear factor erythroid 2-related factor 2, nuclear factor- (NF-) *κ*B, and endothelial nitric oxide synthase and has also been considered to have powerful antioxidant and anti-inflammatory properties. Oxidative stress and inflammation are important pathophysiological mechanisms of various cardiovascular diseases. Therefore, it is not surprising that RSV could exert beneficial effects for the treatment of cardiovascular diseases including IHD [10]. However, some clinical data showed that RSV treatment had little benefit in preventing cardiovascular disease risk factors [11]. Taken together, the precise role and mechanism of action of RSV on IHD remain to be elucidated. Specially, whether high-dose RSV could improve IHD via inhibiting senescence and inflammasome, as suggested recently [12], needs further investigation.

## 2. Materials and Methods

### 2.1. Chemicals

RSV was acquired from Shanghai Winberb Medical S & T Development Co., Ltd. (Shanghai, China). The purity, analyzed by high-performance liquid chromatography, was >99%. RSV is extracted from plants and presents mainly in its “trans” form, which has stronger biological activity than the “cis” form has. RSV must be stored in the dark in both the solid and dissolved states because it can change from the “trans” to the “cis” form in the light.

### 2.2. Animals and Treatments

All experiments procedures were approved by the Institutional Guidelines of Animal Care and Use Committee of Renmin Hospital of Wuhan University and were in strict accordance with the Guide for the Care and Use of Laboratory Animals published by the US National Institutes of Health. All mice were housed in the Cardiovascular Research Institute of Wuhan University (Wuhan, China) at 22°C under a 12 h light/dark cycle with free access to food and water.

Male mice (C57BL/6J, age 9–11 weeks, body weight 26–27.5 g) were purchased from the Institute of Laboratory Animal Science, Chinese Academy of Medical Sciences & Peking Union Medical College (Beijing, China). Classic permanent coronary occlusion without reperfusion was performed to induce myocardial ischemia and myocardial infarction (MI) according to published methods [13]. Briefly, mice were anesthetized with 3% pentobarbital sodium (50 mg/kg, Sigma-Aldrich, St. Louis, MO) by intraperitoneal injection and endotracheal intubated. After adequate anaesthesia and without toe pinch reflex, mice were placed in a supine position on a heating pad to maintain body temperature at 35–36°C. A horizontal cut was made at the fourth intercostal space by cutting the pectoralis muscle transversely to expose the thoracic cage. After opening the pericardium, the left main descending coronary artery (LCA) was located and then ligated with a 6-0 silk suture (3–4 mm). Successful ligation was confirmed when the anterior wall of the left ventricle (LV) turned pale. Mice in the sham group were subjected to the same surgical procedures without LCA ligation. After surgery, the thoracotomy site was closed in layers and mice remained in a supervised setting until fully conscious. Mouse hearts were harvested at the 3^th^, 7^th^, and 14^th^ day according to different experimental design.

According to published protocol to establish the mouse model of ischemia-reperfusion (IR) injury [14], the left anterior descending coronary artery was ligated with a slipknot against PE10 tubing by a 7-0 silk suture. After ischemia for 45 min, the slipknot was disengaged for reperfusion a period of 24 h. And then, mouse hearts were harvested or stained with Evans/TTC staining. The LCA must be ligated again for Evans staining.

RSV is poorly water soluble. Normal saline (NS) was added with carboxymethylcellulose sodium (CMC-Na) to prepare 0.5% CMC-Na solution, and then, the RSV was added into CMC-Na solution, which could provide a homodispersed suspension of RSV. Four groups were included in this study: the RSV (*n* = 15, 320 mg/kg/day) and MI+RSV (*n* = 40, 320 mg/kg/day) groups were treated with RSV 1 week before sham and MI surgery, respectively, and throughout the entire study; the control (*n* = 15) and MI (*n* = 40) groups were treated with an equal volume of normal saline (NS). RSV was dissolved in NS (16 mg/mL) and was administrated by gastric tube at 8 a.m and 5 p.m. The administration dosage of RSV was determined according to a previous study [12]. This dosage (320 mg/kg/d) could provide an effective plasma concentration (10 to 20 *μ*M**)** [12, 15] and was equivalent to patients receiving 150 mg/d of RSV.

### 2.3. Cell Culture

Neonatal rat cardiomyocytes (NRCMs) and cardiac fibroblasts (CFs) were prepared according to our previously reported methods [16]. Briefly, NRCMs and CFs were obtained from 1- to 3-day-old Sprague–Dawley rats through enzymatic separation. NRCMs and CFs were separated by differential adherence culture for 2 h. NRCMs were quantified and cultured at a density of 1 × 10^5^ in 24-well plates or at 5 × 10^5^ in 6-well plates, and Brdu was used to inhibit proliferation of CFs mixed in NRCMs during the first 48 h. CFs were prepared in its three to four passages for the following experiments. Anaero Pack system (Mitsubishi GAS Chemical Co., Inc.) was used to establish hypoxia/reoxygenation (I/R) cellular model. The cardiomyocyte or cardiac fibroblast was exposed to 12 h of hypoxia followed by 24 h of reoxygenation.

Peritoneal macrophages were prepared in our laboratory from adult C57/B6 male mice according to our previously reported methods [17]. Macrophages were cultured in RPMI 160 culture medium containing 15% heat inactivated fetal bovine serum for 1 h. The supernatant was removed, and the adherent cells were peritoneal macrophages. After culture for another 24 h, macrophages were used for experiments.

### 2.4. Analysis of Pressure–Volume Loops

Pressure–volume loop analysis was performed in our laboratory according to published methods [18]. Briefly, mice were anesthetized with 1.5% isoflurane, and then, a microtip catheter transducer (SPR-839, Millar Instruments, Houston, TX) was inserted into the LV to record signals using a pressure–volume system (MPVS-400, Millar Instruments). Heart rate, LV end-diastolic pressure (LVEDP), LV end-systolic pressure (LVESP), LV end-diastolic volume (LVEDV), LV end-systolic volume (LVESV), LV ejection fraction (LVEF), maximal rate of pressure development (*dp*/*dt*_max_), minimal rate of pressure decay (*dp*/*dt*_min_), cardiac output, and stroke work were analyzed using PVAN data analysis software (Millar Instruments).

### 2.5. Histological Analysis

Mouse hearts were harvested and immersed in 10% KCl solution to arrest in diastole. After fixing in 10% formalin for 24 h, hearts were dehydrated and embedded in paraffin and cut transversely close to the apex on 4–5 *μ*m slices. Sirius red in saturated picric acid (PSR) staining was used to evaluate interstitial fibrosis according to published methods [16]. Fibrosis area was assessed by Image-Pro 6.0 (Media Cybernetics, Bethesda, MD). Paraffin sections of the mouse heart were also used for immunohistochemical (IHC) analysis. As described previously [16], after dewaxing, hydration, and antigen retrieval, sections were incubated with primary antibody (p16, 1 : 100, Proteintech, Chicago, IL; p19, 1 : 100, Proteintech; p53 1 : 500, Cell Signaling Technology, Danvers, MA; and NF-*κ*B, 1 : 100, Cell Signaling Technology) at 4°C overnight. The primary antibodies were replaced by phosphate-buffered saline in the negative control group. The following day, sections were incubated with secondary antibody labeled with horseradish peroxidase for 1 h. DAB kit was used for presenting the brownish positive area, which was analyzed by Image-Pro 6.0.

### 2.6. Evans/2,3,5-Triphenyltetrazolium Chloride Staining

For Evans staining, Evans blue was systemically injected into mouse circulation via the orbital vein. After turning the mouse tail tip blue, mice were euthanized, and then the hearts were harvested and kept at −20°C for 30 min. The frozen heart was sectioned into 2 mm short axis slices from the apex to base. The sections were incubated with 1% 2,3,5-triphenyltetrazolium chloride (TTC) solution for 20 min at 37°C. Viable myocardium was red, whereas the infarct area was white [19]. The results were analyzed with Image-Pro 6.0.

### 2.7. Immunofluorescence Staining

Mouse hearts were harvested and embedded at an optimal cutting temperature in compound for frozen sections. NRCMs were fixed with 4% paraformaldehyde for 15 min, and membrane permeability was increased with 0.5% Triton X-100 for 2 min. After antigen retrieval and blocking, sections were incubated with primary antibody at 4°C overnight. The following day, after discarding the primary antibody, sections were incubated with Alexa Fluor® 568 goat anti-rabbit lgG (H+L) (red) or Alexa Fluor® 4888 goat anti-mouse lgG (H+L) antibody for 1 h at 37°C. Slow Fade Gold antifade reagent with DAPI was used for sealing the sections before observation and photography with a fluorescence microscope (Olympus, Tokyo, Japan). Image-Pro 6.0 was used for analysis of the results.

### 2.8. TdT-Mediated dUTP Nick-End-Labeling Staining

The frozen heart sections of mice from the different groups or cultured NRCMs were prepared for TdT-mediated dUTP nick-end-labeling (TUNEL) staining, and a commercial kit (Millipore, Billerica, MA) was used according to the manufacturer's instructions.

### 2.9. SA-*β*-Gal Staining for Senescent NRCMs

NRCMs were cultured on a cover slip in 24-well plates. In the experimental groups, NRCMs treated without or with RSV were cultured in a hypoxic environment for 64 h. In the control groups, NRCMs treated without or with RSV were cultured in normal conditions for 64 h. A commercial kit (C0602, Beyotime Biotechnology, Shanghai, China) were used for SA-*β*-Gal staining according to the manufacturer's instructions.

### 2.10. Western Blot Analysis

Heart tissue or NRCMs were lysed in RIPA buffer containing 20 mM Tris-HCl (pH 7.4), 4% sodium dodecyl sulfate (SDS), and 10% glycerol. The protein lysis sample was boiled for 10 min at 100°C. The bicinchoninic acid protein assay kit was used for quantifying protein concentration. A total of 30 *μ*g protein was electrophoresed in 10% or 12% SDS-polyacrylamide gel electrophoresis and transferred onto polyvinylidene difluoride membranes (Millipore) and then blocked with 5% bovine serum albumin for 1 h. The blots were then incubated with primary antibody overnight. The following day, blots were incubated with secondary antibody labeled with horseradish peroxidase for 1 h after washing three times with Tris-buffered saline with Tween 20. An electrochemiluminescence kit was used for scanning protein blots, which were analyzed by ImageLab 5.2.1 (Bio-Rad Laboratories, Hercules, CA). The following antibodies were used in this study: GAPDH (Cell Signaling Technology, 2118), p16 (Proteintech, 10883-1-AP), p19 (Proteintech, 10272-2-AP), p53 (Cell Signaling Technology, 2524), AC-p53 (Cell Signaling Technology, 2570), Sirt1 (Abcam, Cambridge, UK, ab110304), NLRP3 (Abcam, ab214185), and caspase 1 p20 (Santa Cruz Biotechnology, Santa Cruz, CA, sc-398715).

### 2.11. Quantitative Real-Time Polymerase Chain Reaction

Total RNA and cDNA were prepared according to previously reported methods [16]. Briefly, total RNA was isolated from the heart tissue or NRCMs using an RNA isolation kit (Roche, Mannheim, Germany) and then reverse transcribed into cDNA using the Transcript First-Strand cDNA Synthesis Kit (Roche). PCR amplification was performed by LightCycler 480 with SYBR Green 1 MASTER Mix (Roche). The expression level of target genes was normalized relative to GAPDH. The primers used in this study are shown in Table 1.

### 2.12. Flow Cytometry

Flow cytometry analysis was performed to quantify immune cells (CD45 positive), macrophage (F4/80 positive), and NLRP3. Mouse hearts were subjected to myocardial infarction and treated with or without RSV for 5 days. Hearts were harvested, washed with cold PBS, and then quickly minced into small pieces (1-3 mm^3^) on ice. Heart tissues were digested by 0.1% collagenase II and 2.4 U/mL dispase II in PBS for 30 min at 37°C, and culture medium containing 15% serum was added to terminate cell digestion. Culture medium was filtered and centrifuged with 3000 rpm, and cells were resuspended in PBS. The CD16/32 antibody was used for blocking for 30 min, and cells were incubated with Per-cy5.5-conjugated CD45 (BD Biosciences) and FITC-conjugated F4/80 (BD Biosciences) for 30 min according to the manufacturer's instruction. To stain intracellular NLRP3, cells were fixed and permeabilized by Fixation/Permeabilization Solution (BD Cytofix/Cytoperm™ Plus). After adding the NLRP3 antibody (ABCAM), cells were incubated with Alexa Fluor 633-Goat-Anti-Rabbit-IgG (H-L) secondary antibody for 15 min. A flow cytometer (Beckman, Miami) was used for fluorescence detection, and flowjo_v10 was used for data analysis.

### 2.13. Statistical Analysis

All data were presented as the mean ± standard deviation (SD). SPSS 19.0 (SPSS Inc., Chicago, IL) was used for statistical analysis. the Kaplan-Meier curve followed by log rank test was performed to determine survival rate among different groups. Significance was evaluated using unpaired Student's two-tailed *t*-test or one- or two-way ANOVA. A *p* value < 0.05 was considered significant. All experiments were repeated at least three times independently in cell experiments or were included at least six samples in each group in *in vivo* experiments.

## 3. Results

### 3.1. RSV Improved Cardiac Function after MI

**Figure 1:**
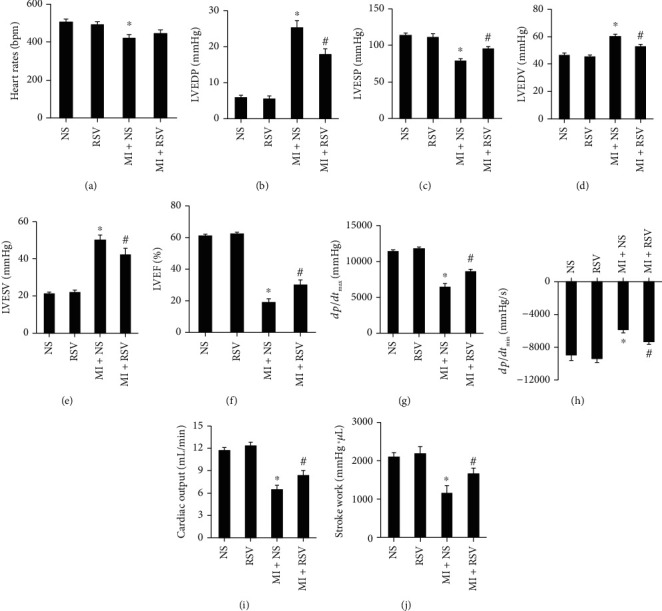
RSV improved mouse cardiac function after MI. Pressure–volume loop analysis showing the (a) heart rate, (b) left ventricle end diastolic pressure (LVEDP), (c) left ventricle end systolic pressure (LVESP), (d) left ventricle end diastolic volume (LVEDV), (e) left ventricle end systolic volume (LVESV), (f) left ventricle ejection fraction (LVEF), (g) *dp*/*dt*_max_, (h) *dp*/*dt*_min_, (i) cardiac output, and (j) stroke work in mice from each group (*n* = 6 in each of the NS and RSV groups, *n* = 7 in each of the MI and MI+RSV groups).Cardiac function was tested at 14^th^ day after MI. Data are presented as the mean ± SD. Two-way ANOVA was performed for significance test. ^∗^*p* < 0.05 vs. the NS group, #*p* < 0.05 vs. the MI+NS group.

Heart rate in the MI group was significantly lower than that in the NS or RSV groups, but no significant difference was observed between the MI and MI+RSV groups (Figure 1(a)). MI caused mouse cardiac dysfunction evidenced by increased LVEDP, LVEDV, and LVESV and decreased LVESP and LVEF (Figures 1(b)–1(f)). Myocardial compliance also declined after MI was evidenced by decreased *dp*/*dt*_max_ and *dp*/*dt*_min_ (Figures 1(g) and 1(h)). Meanwhile, cardiac output and stroke work were markedly reduced in the MI group (Figures 1(i) and 1(j)). All these parameters were improved after RSV treatment (Figures 1(b)–1(j)), and no difference could be detected between the NS and RSV groups (Figures 1(a)–1(j)).

### 3.2. RSV Increased Survival Rate and Decreased Infarcted and Risk Area

**Figure 2:**
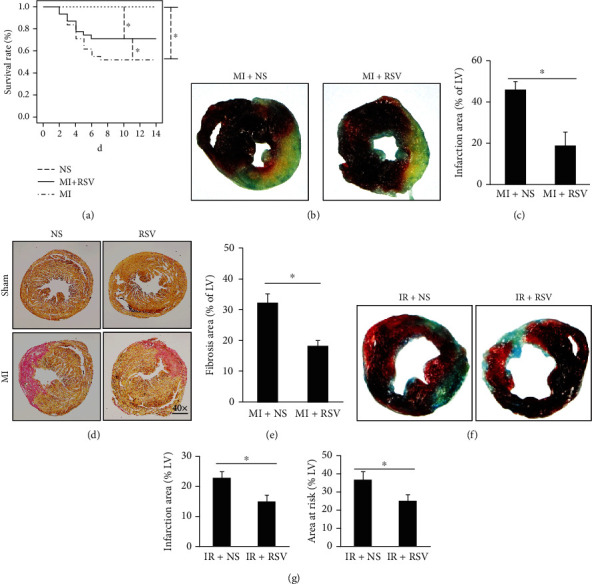
RSV increased survival rate and decreased infarction area. (a) Survival rate after MI surgery (*n* = 10 in each of the NS and RSV groups, *n* = 30 in each of the MI and MI+RSV groups, log rank for significant test), no significance has been tested between the NS and RSV groups, only the curve of the NS group was shown on pictures). (b) Representative Evans blue/TTC (2,3,5-triphenyltetrazolium chloride) staining of heart sections from the MI+NS and MI+RSV groups at the 7^th^ day after myocardial infarction. (c) Quantitative analysis of infarct area in the MI+NS and MI+RSV groups (*n* = 8). (d) Representative PSR staining in the MI+NS and MI+RSV groups at the 7^th^ day after myocardial infarction. (e) Calculated fibrosis area in the left ventricle (LV, *n* = 8). (f) Representative Evans blue/TTC staining of mouse heart sections in the IR+NS and IR+RSV groups at the second day (mouse hearts were ischemic for 45 min and then for 24 h of reperfusion). (g) Quantitative analysis of infarct area and risk area after IR injury (*n* = 7-8). Two-tailed *t*-test was used for significance test. Data are presented as the mean ± SD. ^#^*p* < 0.05 vs. the MI+NS group.

Some previous studies have suggested that RSV could attenuate infarction area in the mouse or rat heart. However, none had shown the effect of RSV on survival rate. As shown in Figure 2(a), RSV treatment obviously decreased mortality after MI surgery. Evans blue/TTC staining was performed to distinguish the infarct area (presented in white) or area at risk (presented in red). Evans blue/TTC staining demonstrated that RSV treatment markedly reduced infarcted area at the 7^th^ day after MI (Figures 2(b) and 2(c)), no significant red area could be identified. PSR staining showed that RSV treatment markedly reduced fibrosis area at the 7^th^ day after MI (Figures 2(d) and 2(e)). Evans blue/TTC staining also presented that RSV treatment markedly reduced infarcted area (white) and area at risk (red) after 45 min ischemia and 24 h reperfusion (Figures 2(f) and 2(g)).

### 3.3. RSV Decreased Expression of Senescence Markers in Mouse Heart

**Figure 3:**
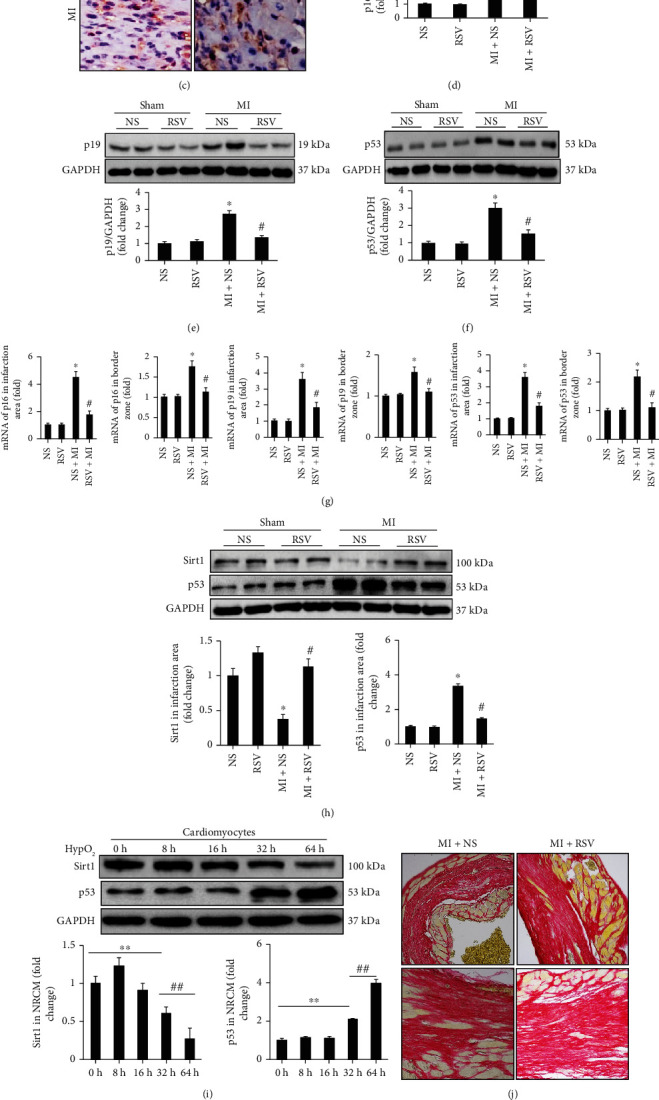
RSV decreased expression of senescence markers in the mouse heart. (a–c) IHC showing the expression of senescence markers including p16, p19, and p53. (d) Representative blots and relative quantitative analysis of p16. (e) Representative blots and relative quantitative analysis of p19. (f) Representative blots and relative quantitative analysis of p53. (g) RT-PCR tested mRNA expression of p16, p19, and p53 in infarction and border zone, respectively. (h) Representative blots and relative expression level of Sirt1 and p53 in mouse heart after 3 days' MI. (i) Representative blots and relative expression level of Sirt1 and p53 in NRCMs at different time points (*n* = 6 in each of the NS and RSV groups, *n* = 6 in each of the MI and MI+RSV groups). (j) PSR staining to examine the collagen deposition. (k) mRNA expression of collagen I/III in the mouse heart tissue. (L) Western blots examined the MMP-2/MMP-9 expression. (m) Relative quantitative expression of MMP-2 and MMP-9. Mouse hearts were harvested for analysis at the 7^th^ day after MI surgery. Data are presented as the mean ± SD. Two-way ANOVA was used for significance test. ^∗^*p* < 0.05 vs. the NS group, ^#^*p* < 0.05 vs. the MI+NS group, ^∗∗^*p* < 0.05 vs. 0 h, ^##^*p* < 0.05 vs. 32 h.

After MI surgery, senescence markers including p16, p19, and p53 were significantly upregulated in the infarction area as shown by both IHC and western blotting experiments (Figures 3(a)–3(f)). With RSV treatment, these senescence markers were obviously downregulated in the infarcted myocardium (Figures 3(a)–3(f)). RT-PCR was performed to investigate these senescence markers in the infarction and border zones, respectively (Figure 3(g)). Ischemia induced the upregulation these markers in both the infarction area and border zone, respectively (Figure 3(g)), which could be effectively downregulated by RSV treatment (Figure 3(g)). We further examined the upstream signal and found that the expression of Sirt1 was significantly decreased accompanied by overt upregulation of p53 (Figure 3(h)). RSV treatment prevented Sirt1 downregulation and p53 upregulation (Figure 3(h)). In NRCMs, hypoxia-treated NRCMs, Sirt1, and p53 presented a similar change as in the *in vivo* experiment in a time-dependent manner, and there was a negative correlation between Sirt1 and p53 expression levels (Figure 3(i)).

A previous study has indicated that p53 inhibition disturbed collagen I/III deposition and contributed to elevated MMP-2 and MMP-9 activities resulted in reduced scar formation and decreased survival rate. Because RSV treatment could inhibit p53 expression and acetylation in this study, we also examined the collagen I/III deposition and MMP2-/MMP-9 expression. Our experiments indicated that RSV-mediated p53 downregulation could not disturbed collagen I/III deposition (Figures 3(j) and 3(k). Western blot analysis presented that MMP-2 and MMP9 were significantly upregulated at the 7^th^ day after MI compared to the control group; however, RSV treatment significantly downregulated the MM2 and MMP9 expression (Figures 3(l) and 3(m)).

### 3.4. RSV Inhibited Inflammasome and Cell Apoptosis in Mouse Heart after MI

**Figure 4:**
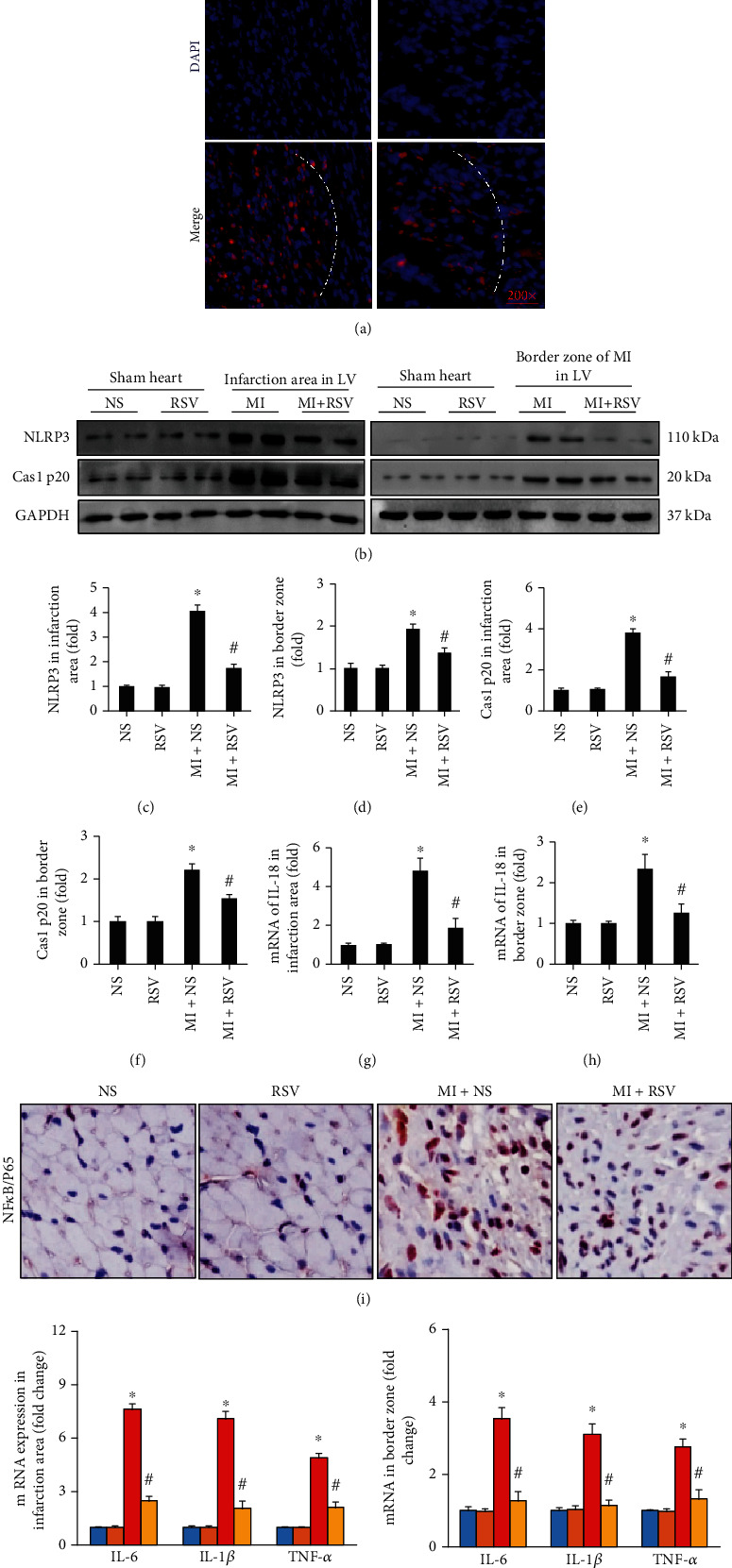
RSV inhibited the inflammasome in the mouse heart after MI. (a) Immunofluorescence showing the distribution of NLRP3 (*n* = 5). (b) Representative blots presented NLRP3 and caspase 1 p20 in infarction area and border zone (*n* = 3). Bar graphs showed (c) NLRP3 in infarction area, (d) NLRP3 in border zone, (e) Cas1 p20 in infarction area, (f) Cas1 p20 in border zone, (g) mRNA level of IL-18 in infarction area, and (h) border zone. (i) IHC staining showing the expression of NF-*κ*B/p65. (j) mRNA expression levels of inflammatory cytokines including IL-1*β*, IL-6, and TNF-*α* in the infarction area and border zone in the mouse heart (*n* = 6 in each of the NS and RSV groups, *n* = 6 in each of the MI and MI+RSV groups). Mouse hearts were harvested for analysis at 7^th^ day after MI. Data are presented as the mean ± SD. Two-way ANOVA was performed to significance test. ^∗^*p* < 0.05 vs. the NS group, #*p* < 0.05 vs. the MI+NS group.

Immunofluorescence was performed to observe the distribution of NLRP3. After MI surgery, massive amounts of NLRP3 were accumulated in the infarction area, whereas a small amount of NLRP3 was scattered in the border zone area (Figure 4(a)). Treatment with RSV significantly decreased the accumulation of NLRP3 both in the infarction area and border zone (Figure 4(a)). MI surgery induced protein overexpression of inflammasome markers (NLRP3 and caspase 1 p20) in the mouse heart tissue, and RSV treatment markedly inhibited the expression of NLRP3 and caspase 1 p20 (Figures 4(b) and 4(c)). Furthermore, the inflammasome-associated inflammatory cytokine IL-18 was significantly upregulated in the infarction area and border zone (Figure 4(d)), which was also suppressed by RSV treatment (Figure 4(d)).

NF-*κ*B/p65 is a key transcript factor in the inflammatory pathway and plays an important role in IHD. IHC staining showed that NF-*κ*B/p65 accumulated in the nucleus after MI, and treatment with RSV clearly blocked the nuclear accumulation of NF-*κ*B/p65 (Figure 4(e)). We further investigated the mRNA expression levels of inflammatory cytokines, including IL-1*β*, IL-6, and tumor necrosis factor- (TNF-) *α*, which were regulated by NF-*κ*B/p65. All these inflammatory cytokines were significantly upregulated in the infarction area and border zone in the mouse heart and were downregulated by RSV treatment (Figure 4(f)).

**Figure 5:**
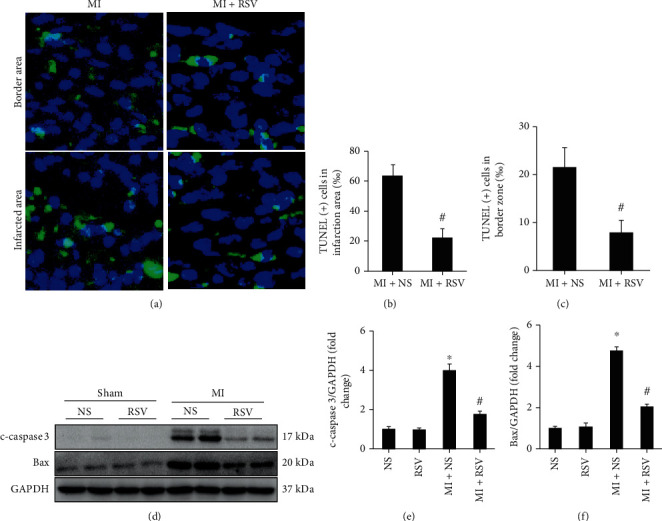
RSV inhibited cell apoptosis in mouse heart after MI. (a) TUNEL staining showing cardiac apoptosis after MI surgery (3 days after MI). (b) Quantitative analysis of TUNEL-positive (+) cells in infarction area. (c) Quantitative analysis of TUNEL-positive (+) cells in border zone of infarction area, five separate fields were calculated independently from 6 samples per group. (b, d) Representative blots and relative expression level of c-caspase 3 and Bax in the mouse heart (*n* = 6). Mouse hearts were harvested at the 3^th^ day after MI surgery. Two-way ANOVA was used for significance examination. Data are presented as the mean ± SD. ^∗^*p* < 0.05 vs. the NS group, #*p* < 0.05 vs. the MI+NS group.

Ischemia and hypoxia induced cardiomyocyte apoptosis. In this study, TUNEL staining demonstrated a high degree of myocardium apoptosis after MI surgery (Figures 5(a)–5(c)), and RSV treatment protected apoptosis (Figures 5(a)–5(c)). Western blot analysis confirmed the antiapoptotic effect of RSV. Apoptosis-associated proteins, cleaved caspase 3 (c-caspase-3) and Bax, were significantly upregulated in the mouse heart after MI, whereas RSV treatment significantly downregulated the expression of c-caspase 3 and Bax (Figures 5(c) and 5(d)).

### 3.5. RSV Prevented NRCM Senescence and Apoptosis Induced by Hypoxia

**Figure 6:**
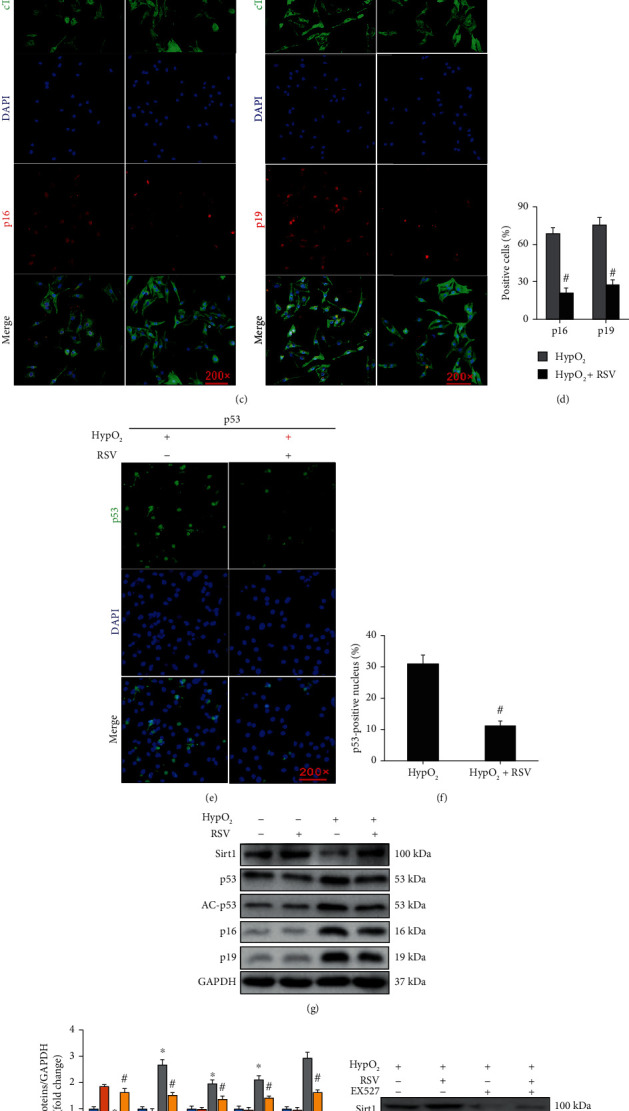
RSV resisted NRCM senescence induced by hypoxia. (a) SA-*β*-Gal staining of NRCMs. (b) Percentage of SA-*β*-Gal-staining-positive cells. (c) Immunofluorescence staining of p16 and p19 in NRCMs. (d) Percentage of p16- and p19-positive cells. (e) Immunofluorescence staining of p53. (f) Calculated percentage of p53-positive nuclei. (g) Representative blots and (h) relative expression levels of Sirt1, p53, AC-p53, p16, and p19 in NRCMs. (h) Relative expression levels of Sirt1, p53, AC-p53, p16, and p19 in NRCMs. (i) Representative blots and (j) relative expression levels of Sirt1, p53, AC-p53, and p19 in NRCMs with or without EX527. Each experiment was repeated three times independently and set up three repeats for each time of experiment. NRCMs were harvested after 64 h or hypoxia or RSV treatment. Data were presented as the mean ± SD. Two-way ANOVA was performed for significance examination ^∗^*p* < 0.05, the HypO2 group vs. the CON or RSV group; #*p* < 0.05, the HypO2+RSV group vs. the HypO2 group.

SA-*β*-Gal accumulated in NRCMs after 64 h of hypoxia treatment, and RSV treatment significantly prevented its accumulation in NRCMs (Figures 6(a) and 6(b)). In addition, the senescence-associated protein markers including p16 and p19 were clearly upregulated in NRCMs and accumulated in the nucleus after hypoxia treatment for 64 h. RSV treatment significantly downregulated p16 and p19 expressions (Figures 6(b)–6(d)).

We found that the Sirt1/p53 pathway might be involved in the regulation of NRCM senescence. p53 accumulated in the nucleus of NRCMs after 64 h of hypoxia treatment (Figures 6(e) and 6(f)), and RSV treatment prevented this accumulation (Figures 6(f) and 6(f)). Western blotting also demonstrated that hypoxia induced the overexpression of p53 in NRCMs via downregulating the expression of Sirt1 (Figures 6(g) and 6(h)). RSV treatment could markedly activate Sirt1 to blunt acetylation of p53 (AC-p53) and inhibit the expression of p53, p16, and p19 (Figures 6(g) and 6(h)). After inhibition of Sirt1by EX527, RSV treatment could no longer inhibit the overexpression of p53 and AC-p53 or the expression of p19 (Figures 6(i) and 6(j)).

**Figure 7:**
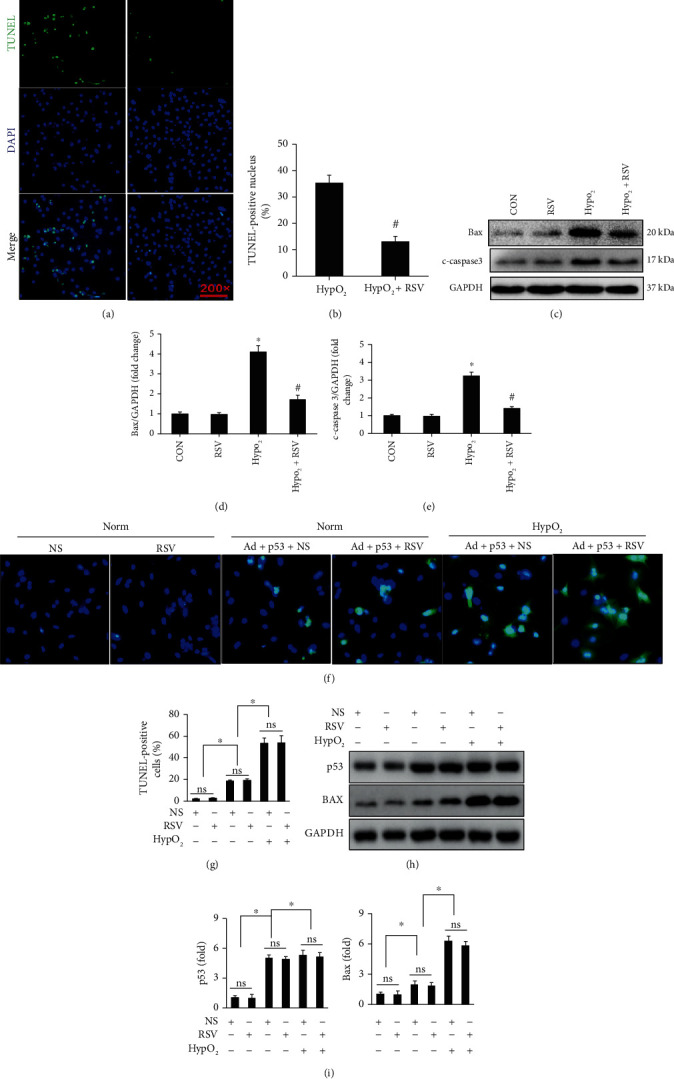
RSV protected against NRCM apoptosis induced by hypoxia. (a) TUNEL staining showing NRCM apoptosis induced by hypoxia. (b) Calculated percentage of TUNEL-positive nuclei. (c–e) Representative blots and relative expression levels of c-caspase 3 and Bax in NRCMs. Each experiment was repeated three times independently. (f) Adenovirus-mediated p53 overexpression contributed to NRCM apoptosis. RSV treatment could not prevent against hypoxia/reoxygenation induced NRCM apoptosis after p53 overexpression. (g) Calculated the ratio of apoptotic NRCM in different groups. (h) Adenovirus-mediated p53 overexpression contributed to Bax overexpression, which could not be inhibited after RSV treatment. (i) Relative quantitative expression of p53 and Bax. Cardiomyocytes were exposed to 12 h of hypoxia followed by 24 h of reoxygenation. Data are presented as the mean ± SD. ^∗^*p* < 0.05 among indicated groups.

Hypoxia treatment obviously induced NCRM apoptosis, which could be prevented by RSV treatment (Figures 7(a) and 7(b)). We also detected the expression of apoptosis-associated proteins in NRCMs. Hypoxia markedly induced the overexpression of c-caspase 3 and Bax in NRCMs, which was blocked by RSV treatment (Figures 7(c)–7(e)). However, p53 overexpression could exaggerate hypoxia/reoxgen-induced NRCM apoptosis (Figures 7(f)–7(i)). RSV treatment could not prevent hypoxia/reoxgen-induced NRCM (Figures 7(f)–7(i)).

### 3.6. RSV Inhibited the Inflammasome in Different Cell Types

**Figure 8:**
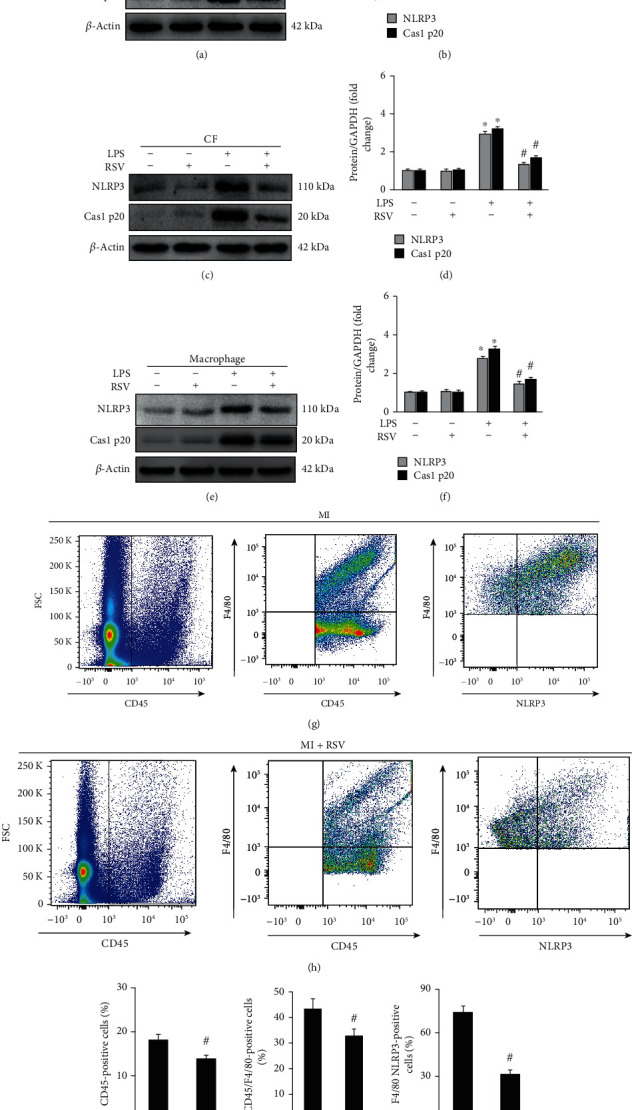
RSV inhibited the inflammasome in different cell types. (a, c, e) Representative blots of NLRP3 and caspase 1 p20 in NRCMs, CFs, and macrophages, respectively. (b, d, f) Relative expression levels of NLRP3 and caspase 1 p20 in NRCMs, CFs, and macrophages, respectively. (g, h) Flow cytometry detected immune cells (marked with CD45), macrophage (marked with F4/80), and NLRP3. (i, j, k) Quantitative analysis for CD45-positive cells, F4/80-positive cells, and double-positive (F4/80 and NLRP3) labeled cells, respectively (*n* = 6). Each experiment was repeated three times independently. Cells were harvested for western blot analysis after 12 h treatment. Data are presented as the mean ± SD. ^∗^*p* < 0.05 vs. the no treatment or RSV group, ^#^*p* < 0.05 the LPS+RSV group vs. LPS group.

The inflammasome forms in CFs [20] and macrophages [21] have been demonstrated. To determine whether RSV treatment inhibited the formation of inflammasome in NRCMs, Rat-CFs, and mouse peritoneal macrophages, these cells were prepared and treated with after lipopolysaccharide (LPS) in vitro experiments. After LPS treatment for 12 h, NLRP3 and caspase 1 p20 were significantly upregulated and RSV treatment prevented the expression of these two inflammasome-associated markers in all three cell types (Figures 8(a)–8(f)). Relying on flow cytometry analysis (Figures 8(g)–8(j)), we showed that RSV treatment could effectively decrease CD45- and F4/80-positive cells (Figures 8(g)–8(j)). Finally, relying on double-positive (F4/80 and NLRP3) labeled macrophage, this study presented that RSV treatment could directly decrease NLRP3 expression in cardiac macrophage.

## 4. Discussion

In this study, we demonstrated the protective effects of RSV on MI in the mouse heart and hypoxia-treated NRCMs, as manifested by decreased mortality and improved cardiac function *in vivo* and a reduction in NRCM apoptosis *in vitr*o. The underlying mechanism may be associated with the regulation of Sirt1-mediated p53 and AC-p53 accompanied by the inhibition of molecular markers of senescence, hence alleviating cardiomyocyte senescence. Furthermore, we revealed that RSV treatment could inhibit the formation of the inflammasome and release of inflammatory cytokines in cardiomyocytes, CFs, and peritoneal macrophages. Our findings were consistent with those from previous studies showing that RSV treatment could protect cardiomyocytes from stress-induced injuries. Nevertheless, the present study provided new insights into the mechanism action of RSV in ischemia- and hypoxia-associated cardiomyocyte injury.

Senescent cells have been reported in the mouse heart under hypoxic and ischemic conditions [6]. In the infarcted mouse heart or hypoxia-treated fibroblasts, p53 was markedly upregulated [6]. Meanwhile, knockdown of p53 by siRNA significantly decreased cell senescence induced by hypoxia [6]. Hypoxia for 48 h in NRCMs could cause the increase of p53 transactivating activity as well as the expression of p21, a well-characterized molecular marker of senescence [5]. Also, the expression of wild-type human p53 was sufficient to induce DNA fragmentation [5]. These studies suggested that activated p53 plays an important role in regulating cell senescence. In the present study, we also observed that p53 was overexpressed in the heart tissue after MI, and hypoxia induced the expression of p53 in a time-dependent manner in NRCMs. RSV treatment could effectively block the expression of p53 and senescence-associated markers.

RSV is a classic agonist of Sirt1, which is a longevity-associated gene that could ameliorate aging-induced LV dysfunction [22]. Impaired nucleocytoplasmic shuttling and activation of Sirt1 during ischemic stress exacerbated ischemia-induced MI [22]. In the senescence-accelerated mouse prone 8 model, RSV attenuated doxorubicin-induced cardiomyocyte senescence and toxicity via restoring Sirt1 activity and depressing USP7-related proapoptotic signaling [23]. In this senescent mouse model, RSV treatment decreased the caspase 3 activity and expression of proapoptotic markers, including p53 and Bax, and apoptotic DNA fragmentation were also significantly reduced in the aged mouse heart after RSV treatment [23]. In doxorubicin-treated mice, RSV supplements could upregulate Sirt1 and strengthen Sirt1-mediated p53 deacetylation to protect against cardiomyocyte apoptosis [24]. Similarly, we observed that ischemia and hypoxia induced the downregulation of Sirt1 in the mouse heart and NRCMs. RSV prevented cardiomyocytes from hypoxia-induced senescence through activating Sirt1. In our *in vitro* experiment, RSV treatment failed to prevent the increase of p53 and senescence marker expression after inhibition of Sirt1 by EX527. Taken together, our results indicate that the protective role of RSV against hypoxia-induced cardiomyocyte senescence was partly associated with the increase of Sirt1-mediated p53 deacetylation. Recently, Ren et al. [25] found that RSV treatment attenuated the development of aging in D-galactose treatment-induced senescent-like cardiomyocytes, by depressing mitochondrial elongation through the activation of Parkin and PINK1. Therefore, it is possible that RSV could attenuate hypoxia- or ischemia-induced senescence through other mechanisms.

IHD is characterized by reduced blood flow and deprivation of oxygen and nutrients, which results in necrotic cells. Cellular necrosis contributes to the release and accumulation of damaged DNA and other cellular debris, which triggers activation of the immune system by damage-associated molecular patterns (DAMPs) [26, 27]. On the one hand, DAMPs could bind to pattern recognition receptors such as Toll-like receptors to initiate an inflammatory response by activating NF-*κ*B/p65 to promote the release of inflammatory cytokines [26, 27], including IL-1*β*, IL-6, and TNF-*α*. On the other hand, DAMPs also activate the nucleotide-binding oligomerization domain-like receptor family [26, 27], which could cause the assembly of inflammasomes to activate caspase 1. Activated caspase 1 could convert pro-1L-1*β* and pro-IL-18 to 1L-1*β* and IL-18, respectively, and finally activate multiple inflammatory pathways ([26]; [30]). Hence, inflammasome formation and activation of NF-*κ*B are undoubtedly key processes involved in IHD and IHD-associated malignant remodeling [3, 4]. In previous studies, RSV has been demonstrated to inhibit NF-*κ*B activation [28, 29], which is consistent with our findings.

In addition to its detrimental effect of causing inflammation-associated injury, NF-*κ*B has been demonstrated to prevent cell apoptosis. Treatment with TNF-*α* in RelA (NF-*κ*B subunit)-deficient mouse fibroblasts and macrophages caused significantly increased cell apoptosis [30]. Mice with RelA deficiency showed embryonic lethality at 15–16 days of gestation because of massive programmed cell death or apoptosis in the liver [31]. These studies demonstrated that the inhibition of NF-*κ*B could increase cell apoptosis. Mechanistically, NF-*κ*B contains elements in its promoters for activating inhibitor of apoptosis protein-1 and X-linked inhibitor of apoptosis protein [32, 33]. Holley et al. also suggested that NF-*κ*B activation might contribute to the expression of the mitochondria-localized antioxidant enzyme manganese superoxide dismutase [34]. However, we believe that the stimulatory function on apoptosis was not inconsistent with the protective effect on inflammation of NF-*κ*B inhibition. NF-*κ*B maintains normal physiological functions through its antiapoptosis effects, but excessive activation of NF-*κ*B induced detrimental effects by producing inflammatory factors. Moreover, almost all studies of ischemia-associated heart injury showed that inhibition of NF-*κ*B activity could attenuate inflammation-associated injury and improve cardiac function.

Here, we first showed that RSV treatment could inhibit the inflammasome in the mouse heart after ischemia-induced MI. The NLRP3 inflammasome has been shown to have a role in the pathophysiology of myocardial injury as consequence of IHD [4]. To date, some studies have suggested that targeting different components of the NLRP3 inflammasome could significantly decrease infarct size and attenuate malignant remodeling [35, 36], but drugs that directly inhibit the NLRP3 inflammasome are still being sought. In the present study, we showed that RSV treatment inhibited the inflammasome after MI evidenced by decreased expression ofNLRP3, pro-caspase 1, and IL-18 *in vivo* and *in vitro*. Consistent with our findings, some studies have also indicated an inhibitory function of RSV treatment on inflammasome-associated inflammation in acute lung injury [37], early brain injury after experimental subarachnoid hemorrhage [38], and chronic kidney disease [39]. Previous studies have shown the source of the NLRP3 inflammasome mainly in CFs [20, 27] and macrophages. Maturation of NLRP3 could cause the release of IL-1*β* and IL-18 from CFs and macrophage, which indirectly cause cardiomyocyte damage. To further demonstrate the effect of RSV on the inflammasome in different cells, we isolated rat CFs, NRCMs, and mouse peritoneal macrophages. After LPS treatment for 12 h, NLRP3 and caspase 1 were significantly upregulated, but treatment with RSV markedly inhibited the production of NLRP3 and caspase 1. These experiments demonstrated that RSV treatment could block the inflammasome-associated inflammatory response in different cell types.

## 5. Conclusion

In summary, our findings demonstrated that RSV treatment was able to ameliorate cardiomyocyte injury induced by ischemia or hypoxia. This study extended the understanding of the protective effect of RSV on the mouse heart and cardiomyocytes against ischemia- or hypoxia-associated apoptosis and injury via regulating cell senescence and inhibiting the inflammasome. Thus, RSV might be a potential drug for treatment or auxiliary therapy for IHD as a result of its multiple biological functions. Clinical studies on the application of RSV are needed to confirm and elaborate the protective effects of RSV in clinical treatment. Besides, our study also raised questions whether there were some mechanism association between RSV's antisenescence and anti-inflammasome. A previous study [40] has presented that NAD^+^ reduction in acute pancreatitis contributed to augmented inflammasome signaling accompanied with decreased Sirt1 expression and increased AC-p53 [40]. However, pharmacological stimulation of NQO1 could restore NAD^+^ level resulted in alleviated inflammasome signaling accompanied with increased Sirt1 expression and decreased AC-p53 [40]. So, it seemed to be reasonable to deduce that decreased inflammasome signaling by RSV treatment in this manuscript could also be involved in the Sirt1/p53 pathway. However, the exact relationship between antisenescence and anti-inflammasome of RSV remains to be clarified in future experiments. Finally, we must note that senescence reflects inhibition of uncontrolled replication in proliferative cells; however, adult cardiomyocytes are terminally differentiated cells without proliferation; the roles and mechanisms of adult cell senescence needed more elaborate investigation. Besides, NRCMs and adult cell possess different fundamental mechanisms in regulating their respective pathophysiological activity, so it should be cautious to extrapolate results (cell senescence) obtained in the NRCM to adult mouse hearts.

## Figures and Tables

**Table 1:** Primers used for RT-PCR.

Genes	Forward	Reverse
GAPDH	ACTCCACTCACGGCAAATTC	TCTCCATGGTGGTGAAGACA
IL-1*β*	CCGTGGACCTTCCAGGATGA	GGGAAGGTCACACACCAGCA
IL-6	AGTTGCCTTCTTGGGACTGA	TCCACGATTTCCCAGAGAAC
TNF-*α*	CATCTTCTCAAAATTCGAGTGACAA	TGGGAGTAGACAAGGTACAACCC
IL-18	GACAGCCTGTGTTCGAGGAT	TCCTTCACAGAGAGGGTCACA
Collagen I	TGGTACATCAGCCCGAAC	GTCAGCTGGATAGCGACA
Collagen III	GTCAGCTGGATAGCGACA	GAAGCACAGGAGCAGGT GTAGA

## Data Availability

If someone or any research requests data or any details of the experiment about this article, please contact the corresponding author (Hai-han Liao, email address:liaohaihan@whu.edu.cn).
